# P014. Migraine and hypnosis

**DOI:** 10.1186/1129-2377-16-S1-A86

**Published:** 2015-09-28

**Authors:** Marco Ardore, Lorenzo Pinessi, Lidia Savi

**Affiliations:** Department of Oncology, Oral Medicine and Oral Oncology Unit, University of Turin, Orbassano, Italy; Department of Neurosciences, University of Turin, Turin, Italy; Headache Centre, Neurology I, Department of Neurosciences, University Hospital City of Health and Sciences, Turin, Italy

## Aims

Hypnosis is an alternative therapy treatment for those patients who do not want pharmacological therapy or for those who need more therapy to control pain due to migraine attacks. In our study, we evaluated 105 migraine patients randomly selected from those patients treated at the Headache Centre of the University Hospital City of Health and Sciences of Turin and who had shown interest in hypnotherapy, had been diagnosed according to the ICHD-3beta criteria, and had experienced headache days (HD) in the last three months.

## Methods

We submitted a questionnaire reporting a brief description of hypnosis, proposing the possibility to try this therapy. We obtained informed consent from the patients interested in the study. At a later date, we gathered diagnosis and HD from the patients’ clinical records.

## Results

Patients were divided by gender: 50% males and 55% females were responders with a mean of 53%. According to the ICHD diagnostic criteria: 42% of patients with migraine without aura were interested in hypnosis (HI) and 35% were not interested in hypnosis (NHI); migraine with aura: 1% (HI) and 2% (NHI); migraine without and with aura: 7% (HI) and 6% (NHI); chronic migraine: 4% (HI) and 4% (NHI). Analysing HD we found that: 65% of patients with < 3 HD were not interested in trying hypnotic therapy (HT) for migraine treatment; patients with > 30 HD, 78% were interested; patients with > 11 HD, 68% were interested in HT and 32% were not; in patients with < 11 HD, 61% were not interested in HT and 39% showed interested.

## Conclusions

We have for the first time an estimate of migraine patients interested in HT regardless of headache diagnosis and gender. There is a 53% probability that a migraine subject could be interested in HT. Taking HD into account, we found a linear correlation between HD and treatment seeking. Patients with > 12 HD or < 12 HD have a 2/3 probability of being or not being interested in HT.

Written informed consent to publication was obtained from the patient(s).

